# 

**Published:** 1891-09-12

**Authors:** 


In the "Lancet" of Nov. 11, 18[illegible], BARON LIEBIG says: [illegible] price with a preparation of meat, combining in itself the albuminous together with the extractive principles, such a preparation would have to be preferred to the Extractum Carnis, for it would contain all the nutritive constituents of meat." Again—"I have before stated that in preparing the Extract of Meat, the Albuminous principles remain in the residue, they are lost to nutrition, and this is certainly a great disadvantage."

**BOVRIL**

"BOVRIL" contains the albumen and fibrine in the most perfect possible form, and to [illegible]o know the requirements of the human system and the constituents of food, it will be apparent that this albumen and [illegible] identical with what the body requires for recuperation, and that as a perfect form of concentrated nourishment it must [illegible]e any animal aliment at present known.

SOLD THROUGHOUT THE UNITED KINGDOM.

### THE HOSPITAL

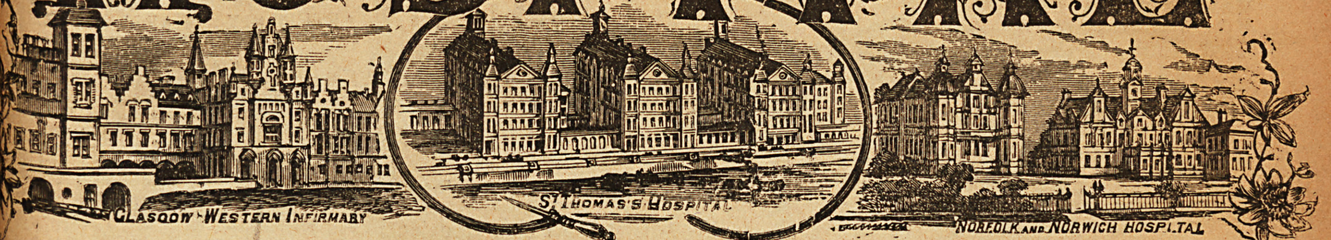

WITH EXTRA NURSING SUPPLEMENT

No. 259. Vol. X.] SATURDAY, SEPTEMBER 12th, 1891. [ONE PENNY.

